# Myc Promoter-Binding Protein-1 (MBP-1) Is a Novel Potential Prognostic Marker in Invasive Ductal Breast Carcinoma

**DOI:** 10.1371/journal.pone.0012961

**Published:** 2010-09-23

**Authors:** Mariavera Lo Presti, Arianna Ferro, Flavia Contino, Claudia Mazzarella, Silvia Sbacchi, Elena Roz, Carmelo Lupo, Giovanni Perconti, Agata Giallongo, Paola Migliorini, Antonio Marrazzo, Salvatore Feo

**Affiliations:** 1 Dipartimento di Oncologia Sperimentale e Applicazioni Cliniche, Università di Palermo, Palermo, Italy; 2 Istituto di Biomedicina e Immunologia Molecolare, CNR, Palermo, Italy; 3 Dipartimento di Medicina Interna, Università di Pisa, Pisa, Italy; 4 Dipartimento Oncologico di III livello La Maddalena, Palermo, Italy; Institut de Génomique Fonctionnelle de Lyon, France

## Abstract

**Background:**

Alpha*-*enolase is a glycolytic enzyme that catalyses the formation of phosphoenolpyruvate in the cell cytoplasm. α-Enolase and the predominantly nuclear Myc promoter-binding protein-1 (MBP-1) originate from a single gene through the alternative use of translational starting sites. MBP-1 binds to the P2 c-myc promoter and competes with TATA-box binding protein (TBP) to suppress gene transcription. Although several studies have shown an antiproliferative effect of MBP-1 overexpression on several human cancer cells, to date detailed observations of α-enolase and MBP-1 relative expression in primary tumors versus normal tissues and their correlation with clinicopathological features have not been undertaken.

**Methodology and Findings:**

We analyzed α-enolase and MBP-1 expression in normal breast epithelium and primary invasive ductal breast carcinoma (IDC) from 177 patients by Western blot and immunohistochemical analyses, using highly specific anti-α-enolase monoclonal antibodies. A significant increase in the expression of cytoplasmic α-enolase was observed in 98% of the tumors analysed, compared to normal tissues. Nuclear MBP-1 was found in almost all the normal tissues while its expression was retained in only 35% of the tumors. Statistically significant associations were observed among the nuclear expression of MBP-1 and ErbB2 status, Ki-67 expression, node status and tumor grade. Furthermore MBP-1 expression was associated with good survival of patients with IDC.

**Conclusions:**

MBP-1 functions in repressing c-myc gene expression and the results presented indicate that the loss of nuclear MBP-1 expression in a large number of IDC may be a critical step in the development and progression of breast cancer and a predictor of adverse outcome. Nuclear MBP-1 appears to be a novel and valuable histochemical marker with potential prognostic value in breast cancer.

## Introduction

Breast cancer development is the result of an accumulation of molecular abnormalities that activate oncogenes and inactivate tumor suppressor genes. Understanding the biology of the disease is critical for developing novel strategies for early detection, prevention, classification and treatment.

Enolase was originally characterized as an enzyme involved in glucose metabolism. Three enolase isoforms have been found in mammalian cells, they are named α- (ENO1), β- (ENO3), and γ- (ENO2) enolase. The expression of these isoforms is developmentally regulated in a tissue-specific manner: the ENO1 gene product is widely distributed in a variety of tissues, whereas the ENO2 and ENO3 products are primarily found in neuron/neuroendocrine and muscle tissues, respectively. Enolase forms homodimers or heterodimers to convert 2-phosphoglycerate into phosphoenolpyruvate in the glycolytic pathway. More recent evidence, however, indicates that α-enolase is a multifunctional protein [1]. In addition to its glycolytic function, which is primarily exerted in the cytoplasm, α-enolase has been found on the cell surface, where it functions as a plasminogen receptor, implying that membrane-bound enolase may play a role in inflammation and tissue invasion [2]. In hypoxic situations, α-enolase also acts as a stress protein and it has been speculated that its up-regulation may provide protection to cells by increasing anaerobic metabolism [3].

Changes in ENO1 gene expression have been observed in several cancer cell models, whereas the clinical correlation of ENO1 expression to tumor status has not yet been clearly defined. Up-regulation of ENO1 has been reported in several highly tumorigenic or metastatic cell lines derived from alveolar type II pneumocytes [4], small cell lung cancer [5], head and neck cancers [6]. Similarly, studies examining enzymatic activities in breast cancer indicated a role for α-enolase in tumor progression [7]. Two recent studies, one reporting the overexpression of α-enolase in 18 out of 24 different types of cancer, including breast cancer [8], the other demonstrating the association of phosphorylated isoforms with pancreatic ductal adenocarcinoma [9], further support a correlation between ENO1 expression and its general pathophysiological role in cancer formation. By using an alternative translation start codon, ENO1 transcripts, which encode the full length polypeptide of about 48 kDa, can also be translated into a shorter protein (37 kDa), called Myc promoter-binding protein-1 (MBP-1) [10], [11], which acts as a negative regulator of c-myc gene expression by interacting with the major promoter [12], [13]. So far, several MBP-1-associated proteins have been identified, including histone deacetylase HDAC1 [14], MIP2A/sedlin [15], [16], MEK5α [17], NS1-BP [18], and the Notch1 receptor intracellular domain [19]. The target genes of MBP-1 transcriptional activity remain unclear except for c-myc and, as recently reported, COX2 [20]. It has been reported that MBP-1 may regulate target genes at least in part through the p53–p21 pathway [21]. Mounting evidence indicates that both α-enolase and MBP-1 might be involved in tumorigenesis of breast carcinoma [22], non-small cell lung cancer [23], [24], hepatitis C virus–related hepatocellular carcinoma [25], prostate tumor [17], [26], squamous cell carcinoma [27] and neuroblastoma [28]. It has also been shown that MBP-1 expression reduces the invasive ability of breast cancer cells both *in vitro* and in a mouse model [22], [29], and α-enolase may participate in the control of epithelial to mesenchymal transitions [30]. While the role of the exogenous expression of MBP-1 in transcription and cell growth regulation appears to be established, the *in vivo* function of this protein is poorly understood. Detailed observations of α-enolase and MBP-1 expression and localization in human breast epithelium and breast carcinomas have not been undertaken thus far.

In the present study, by using highly specific monoclonal antibodies to α-enolase, we confirm that ENO1 is significantly overexpressed in primary breast tumor specimens. Furthermore, we report for the first time the characterization of MBP-1 expression in primary breast cancers of different histological grades from patients with various clinicopathological features. Immunohistochemical and Western blot analyses indicate that nuclear expression of MBP-1 inversely correlates with the prognosis of disease recurrence for patients with IDC. Using multivariate analysis, the effectiveness of nuclear MBP-1 expression as a prognostic factor was assessed. Our results strongly suggest that MBP-1 could be an independent biomarker for the prediction of breast cancer progression and/or outcome.

## Materials and Methods

### Patients information and tissue specimens

Women with histological verified IDC, treated at the Departments of Surgery and Oncology, La Maddalena Hospital, Palermo, from 2001 to 2008 were included in the study. Patients with in situ carcinoma, distant metastases at the time of the diagnosis, synchronous or metachronous bilateral breast cancer, malignancy other than breast cancer in history, and women who did not undergo breast surgery were excluded. The 177 patients, of whom sufficient clinical data and histologic specimens could be retrieved, were chosen for the analysis. Tumor tissues were submitted to routine histopathologic examination. Among the 177 patients, 79 were followed up for the first 5 years after the diagnosis at 6-month intervals. The median age at the time of the diagnosis was 59 years (range 31–83 years). The median duration of the follow-up of the patients still alive was 5.1 years (range 2.5–6.1 years) and of all the patients 4.0 years (range 0.3–6.1 years). Representative clinical and histological data of the IDC patients are summarized in Table 1. All experiments using human tissues were performed with the written patients' informed consent and with the approval of Institutional Review Boards from La Maddalena Hospital.

**Table 1:** Correlation between MBP-1 expression and clinicopathological characteristics of breast cancer patients.

Variable	Total (%)	MBP-1 nuclear immunoreactivity		*P*
		Positive (%)	Negative (%)	
**Age (yr)**				
≤50	51 (29)	15 (23)	36 (32)	
>50	126 (71)	51 (77)	75 (68)	NS
**Tumor size**				
≤2 cm	68 (38)	28 (42)	40 (36)	
>2 cm	109 (62)	38 (58)	71 (64)	NS
**Node status**				
negative	85 (48)	44 (67)	41 (37)	
positive	92 (52)	22 (23)	70 (63)	0.0002
**Tumor grade**				
1–2	80 (45)	43 (65)	37 (33)	
3–4	97 (55)	23 (35)	74 (67)	<0.0001
**ErbB2**				
negative	122 (69)	58 (88)	64 (58)	
positive	55 (31)	8 (12)	47 (52)	<0.0001
**ER**				
negative	39 (26)	15 (59)	24 (25)	
positive	112 (74)	41 (61)	71 (75)	NS
**PG**				
negative	57 (38)	16 (29)	41 (43)	
positive	94 (62)	40 (71)	54 (57)	NS
**Ki67**				
negative	33 (19)	19 (29)	14 (13)	
positive	144 (81)	47 (71)	97 (87)	0.0096
**Recurrences**				
recurrent	32 (40)	4 (17)	28 (51)	
non recurrent	47 (60)	20 (83)	27 (49)	0.0057
**Death for disease**				
Yes	19 (24)	3 (13)	16 (29)	
No	60 (76)	21 (87)	39 (71)	NS

**NOTE**: Statistical analyses were done by the Fisher's exact test. A *P* value of <0.05 was considered significant.

Abbreviations: ER, estrogen receptor; PG, progesterone receptor;

NS, nonsignificant.

### Antibodies and Western Blot analysis

Anti-α-enolase monoclonal antibodies (mAbs) were generated by immunizing mice with a GST-enolase chimeric peptide as described previously [31]. Two mAbs specific to the N-terminus (ENO-276/3) and the C-terminus (ENO-19/8) of human α-enolase were affinity-purified on ProtA-Sepharose 4B (Amersham Biosciences, Sweden). The affinity-purified anti-α enolase mAbs were used for immunohistochemical and Western blot analysis. Anti-c-Myc antibody (sc-40) was purchased from Santa Cruz (Santa Cruz Biotechnology, CA), rabbit anti-Flag antibody (M2) was from Sigma (Sigma Chemical Company, St Louis, MO).

For Western blot analysis frozen tissue (about 100 mg) was homogenized in 500 µl of ice-cold RIPA buffer (50 mM Tris pH 7.4, 150 mM NaCl, 1% Triton X-100, 0.1% SDS, 1% sodium deoxycholate, 1 mM EDTA, 0.5 mM DTT) with freshly added protease and phosphatase inhibitors (Sigma Chemical Company, St Louis, MO). After 30 min incubation on ice, samples were spun at 12.000 rpm for 20 min at 4°C and supernatants were collected. Human embryonic kidney 293T cells were transiently transfected with Flag-Eno and Flag–MBP-1 expressing constructs as previously reported [18]. Total cell lysates were prepared by directly harvesting cells in RTB buffer (8 M Urea, 2 M Thiourea, 4% CHAPS, 100 mM DTT) supplemented with protease and phosphatase inhibitors. Transfection efficiency (about 80%) was assessed in parallel cultures by the use of a vector expressing β-galactosidase and protein extracts were screened for the presence of recombinant enolase breakdown products by preliminary analysis with anti-Flag antibodies. Only lysates with no detectable breakdown products were used for further analysis.

Protein concentrations from tissue and cell lysates were determined by the Bradford protein assay (BioRad, Hercules, CA). Aliquots corresponding to 30 µg of samples were separated on 4-12% polyacrylamide gradient gels (Invitrogen, Carlsbad, CA), and electrotransferred to PVDF membrane (Amersham Biosciences, Sweden), according to manufacturer's instructions. Membranes were probed with primary antibodies and horseradish peroxidase-conjugated secondary antibodies (Amersham Bioscience, Sweden). To ensure equal loading of protein among samples, membranes were additionally probed with β-actin antibody (Sigma Chemical Company, St Louis, MO). Detection was performed with chemiluminescent substrates (Pierce Biotechnology, Rockford, IL) and densitometric analysis was used to quantify signals.

### RNA extraction, reverse transcription, and real-time PCR

Total RNA from breast tumor samples was extracted using Trizol reagent (Invitrogen, Carlsbad, CA) according to the manufacturer's instructions. RNA was reverse-transcribed into cDNA by using Superscript II reverse transcriptase (Invitrogen, Carlsbad, CA) as described previously [32]. Amplification reactions were performed in a 25 µl reaction volume containing about 20 ng total RNA equivalents, 20 pmoles of c-myc specific primers (SABioscience, Frederick, MD) and the Power SYBER Green PCR ready-mix using a 7300 thermal cycler (Applied Biosystems, Foster City, CA). PCR conditions were: denaturation at 95 C° for 3 minutes, followed by 35 cycles 95 C° for 20 seconds, 60 C° for 15 seconds, and 72 C° for 15 seconds, and a final extension at 72°C for 7 minutes. Reaction specificity was controlled by post-amplification melting curve analyses and agarose gel electrophoresis of the amplified products. To correct for the experimental variations between samples, Ct value of TATA-binding protein (TBP) mRNA, amplified with specific primers (SABioscience, Frederick, MD), was determined in each PCR reaction. All data shown were generated from three independent experiments and are expressed as mean ± SD.

### Immunohistochemistry

Immunohistochemistry was performed on BR804 Breast Tumor Tissue Array (US Biomax Inc., Rockville, MD, USA), and on tissue sections from archived formalin-fixed, paraffin-embedded tissue blocks from patients. For all samples, α-enolase, estrogen receptor (ER), progesterone receptor (PR), human epidermal growth factor receptor 2 (ErbB2) and Ki67 status were assessed by immunohistochemistry. From each individual paraffin-embedded block serial sections of 3 µm were cut, deparaffinized in xylene and hydrated in a graded series of alcohol. After antigen retrieval in citrate buffer, staining was performed using the BenchMark automated staining system (Ventana Medical System, Tucson, AZ, USA) with primary antibodies against ER-α (SP1, dilution 1∶500), PR (1E2, dilution 1∶500), Ki67 (30-9, dilution 1∶500), c-ErbB2 (4B5, dilution 1∶500), α-enolase affinity purified monoclonal antibodies (mAbs) (ENO-19/8 and ENO-276/3, 1.0 µg/ml). Rabbit anti-human polyclonal antibodies were used as a negative control (dilution 1∶500). In this study, ER, PR and Ki67 staining were scored as positive when more than 10% of tumor cells showed staining. Tumors were graded as ErbB2-positive with a score of 3+ and negative with a score of 0 or 1+, according to common pathological guidelines. Tumors ErbB2-positive 2+ were evaluated by in situ hybridization (FISH) with a dual-color probe (PathVysion ErbB2/CEP17; Vysis, Downers Grove, IL, USA), according to manufacturer's instructions, and scored positive when ErbB2 gene amplification was found.

An immunohistochemical grading scale for α-enolase expression into the cytoplasm and nucleus, ranging from none (0) to weak (1), or moderate (2) to strong (3), was empirically determined. In addition, for nuclear staining, the percentage of cells labeling was graded as negative for ≤20%, or positive for >20%. To confirm the specificity of the α-enolase-staining serial sections of a subset of the specimens (n = 12) were stained using antibodies preadsorbed for 1 hour at room temperature with the antigenic peptides for mAbs ENO-276/3 or the recombinant α-enolase-GST polypeptide [31] for mAbs ENO-19/8. The staining intensity of normal breast glands for a given patient was assessed from sections of margin tissue blocks or from morphologically-identified normal glands within the same slide containing malignant tumors. Normal mammary tissues identified either adjacent to the tumor cells, and/or on corresponding margin tissue sections, were analyzed in 158 cases. Slides were visualized with light microscopy and qualitatively scored while blinded to clinicopathological variables.

### Statistical Analysis

A database containing α-enolase and MBP-1 status and relevant covariables was assembled and analyzed using GraphPad Prism version 4.02 for Windows (GraphPad Software, Inc. La Jolla, CA, USA). The association between MBP-1 expression and molecular and clinicopathological variables was assessed using contingency table methods and tested for significance using the Fisher's exact test. Correlations were determined using the non-parametric Spearman's test. Life tables were computed according to the Kaplan-Meier method. Distant disease-free survival was calculated from the date of the diagnosis to the occurrence of metastases outside the regional area or to death from breast cancer. Patients who died from intercurrent causes were censored at the date of death. Survival curves were compared with the Cox-Mantel log rank test. The significance of various variables for survival was analyzed by the Cox proportional hazards model in the multivariate analysis. All tests of statistical significance were two-tailed, *p*-values less than 0.05 were considered statistically significant.

## Results

### α-Enolase but not MBP-1 is overexpressed in the majority of human breast tumors

**Figure 1:**
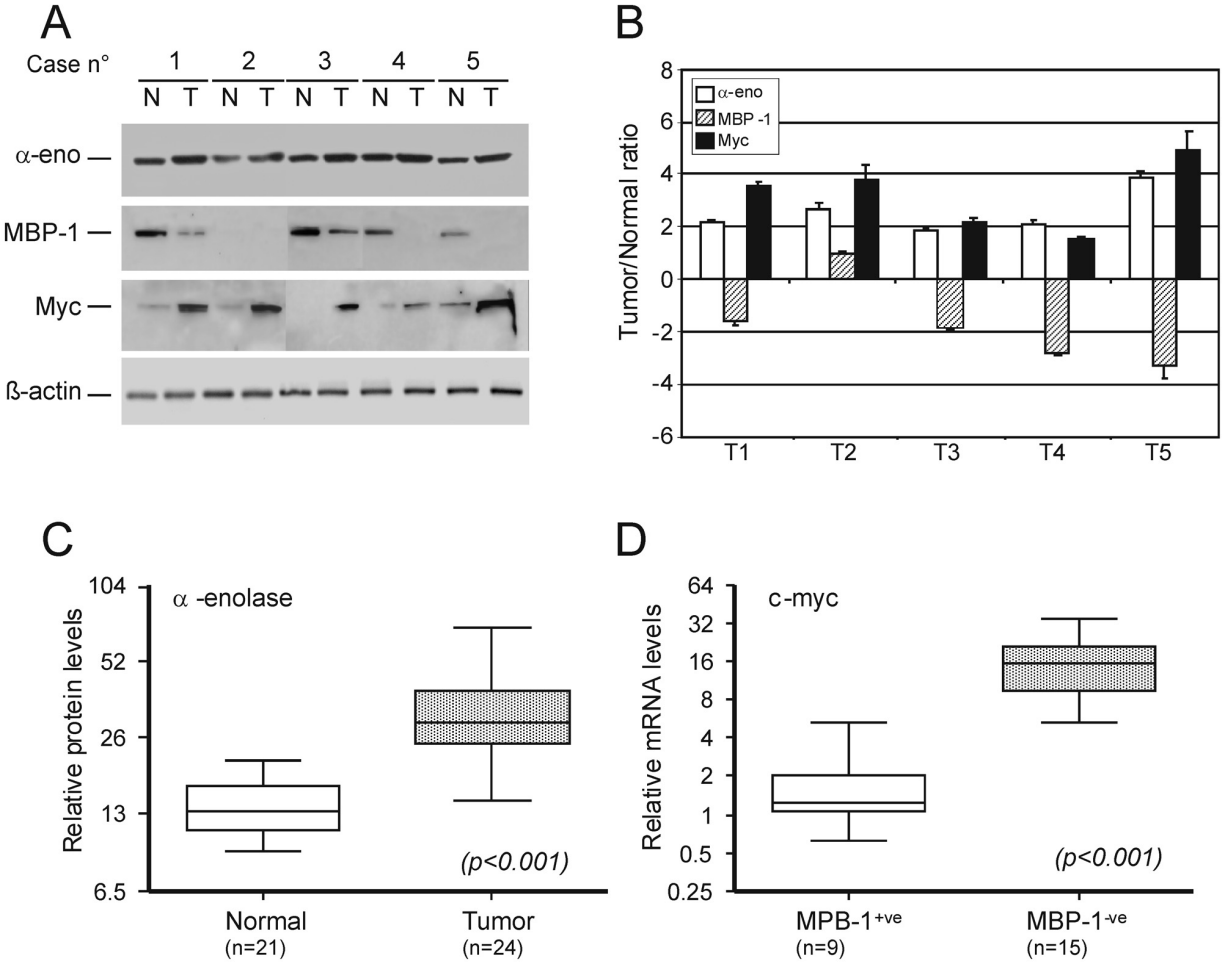
Relative expression of α-enolase, MBP-1 and Myc in primary breast tumors and adjacent normal tissue. **A**. Representative Western blot analysis of α-enolase (α-eno), MBP-1, Myc and β-actin proteins in total lysates (30 µg) from breast tumors (T) and normal-matched tissues (N) was performed as described in materials and methods. **B**. Graphic representation of the averaged tumor/normal (T/N) ratios of α-enolase (white bars), MBP-1 (striped bars) and Myc (black bars) protein expression quantified by the densitometric analysis of Western blot results. Expression levels were normalized to β-actin. Columns are the mean of three parallel experiments; bar, ±SD. **C**. α-Enolase protein levels in normal and breast cancer tissues. The Box plot represents the α-enolase/β-actin ratio determined in 24 breast tumors and 21 normal-matched tissues. Significantly higher levels of α-enolase were present in breast cancers than normal tissues (t-test value, *p*<0.001). **D**. Myc mRNA expression levels in normal and breast cancer tissues. Transcripts were analyzed by real-time PCR and normalized with respect to TBP mRNA. Box plot of c-myc mRNA levels in MBP-1-positive (+^ve^) and negative (−^ve^) breast tumors. Myc mRNA levels are significantly associated with MBP-1 status (t-test value *p*<0.001). In C and D bars above and below the boxes represent the maximum and minimum expression. Each box delineates the first to third quartiles of expression, and the central bar represents the median.

To evaluate the levels of α-enolase (48 kDa) and MBP-1 variant (37 kDa) expression in a set of tumor samples with paired normal breast tissues from 24 patients with IDC, Western blot analysis was performed using a previously described monoclonal antibody (ENO-19/8) which recognises both proteins [18]. Expression was correlated to Myc protein levels in the same samples. A representative Western blot analysis is shown in Figure 1A, while the averaged tumor/normal tissue ratios of α-enolase, MBP-1 and myc protein levels for the same samples, after normalization for β-actin expression, are graphically represented in Figure 1B.

All normal breast tissues showed moderate expression of α-enolase, whereas stronger expression was observed in the paired tumor samples; overall MBP-1 displayed an opposite pattern of expression and, as expected, higher levels of Myc protein were detected in tumors compared to normal tissues.

Statistical analysis of Western blot data confirmed the presence of significantly higher levels of α-enolase protein in breast cancer samples than in normal tissues (*p*<0.001, Figure 1C).

MBP-1 has been reported to act as a c-myc gene transcriptional repressor; therefore we investigated c-myc mRNA level in the same samples by quantitative reverse transcription polymerase reaction (qRT-PCR). The relative mRNA levels of c-myc showed a significant association with MBP-1 protein status (*p*<0.001) and the median c-myc expression was about nine times less in tumors that retained MBP-1 than in those that did not (Figure 1D).

All together these results, in line with those previously reported, further suggest that repression of c*-*myc is exerted by the alternatively translated MBP-1 protein rather than α-enolase, although both proteins can bind to the c-myc promoter [10], [11].

### Enolase nuclear immunoreactivity and MBP-1 expression

To further investigate the significance of α-enolase and MBP-1 expression in breast cancer, we firstly analyzed protein expression in a series of patients with IDC using a commercially available tumor tissue array (TMA) and the ENO-19/8 monoclonal antibody.

The 35 tumor samples and adjacent normal breast tissues, present in the TMA, exhibited various intensities of nuclear and/or cytoplasmic staining; strong cytoplasmic staining confirmed that higher levels of α-enolase are present in cancer lesions than in surrounding tumor-adjacent normal tissues, as shown by Western blot analysis (Figure 1A–C).

Interestingly, nuclear staining was detected in 12 out of 35 (34%) tumor samples, whereas nuclear staining of moderate to strong intensity was detected in almost all (97%) the adjacent normal mammary tissues.

Studies conducted so far on tumor samples and normal tissues rely on the use of commercially and non-commercially available antibodies recognizing both α-enolase and MBP-1, this has hampered the possibility to clearly evaluate the relative expression of the two proteins by immunohistochemistry. This observation prompted us to further characterize a number of monoclonal antibodies we have previously generated against α-enolase [31].

**Figure 2:**
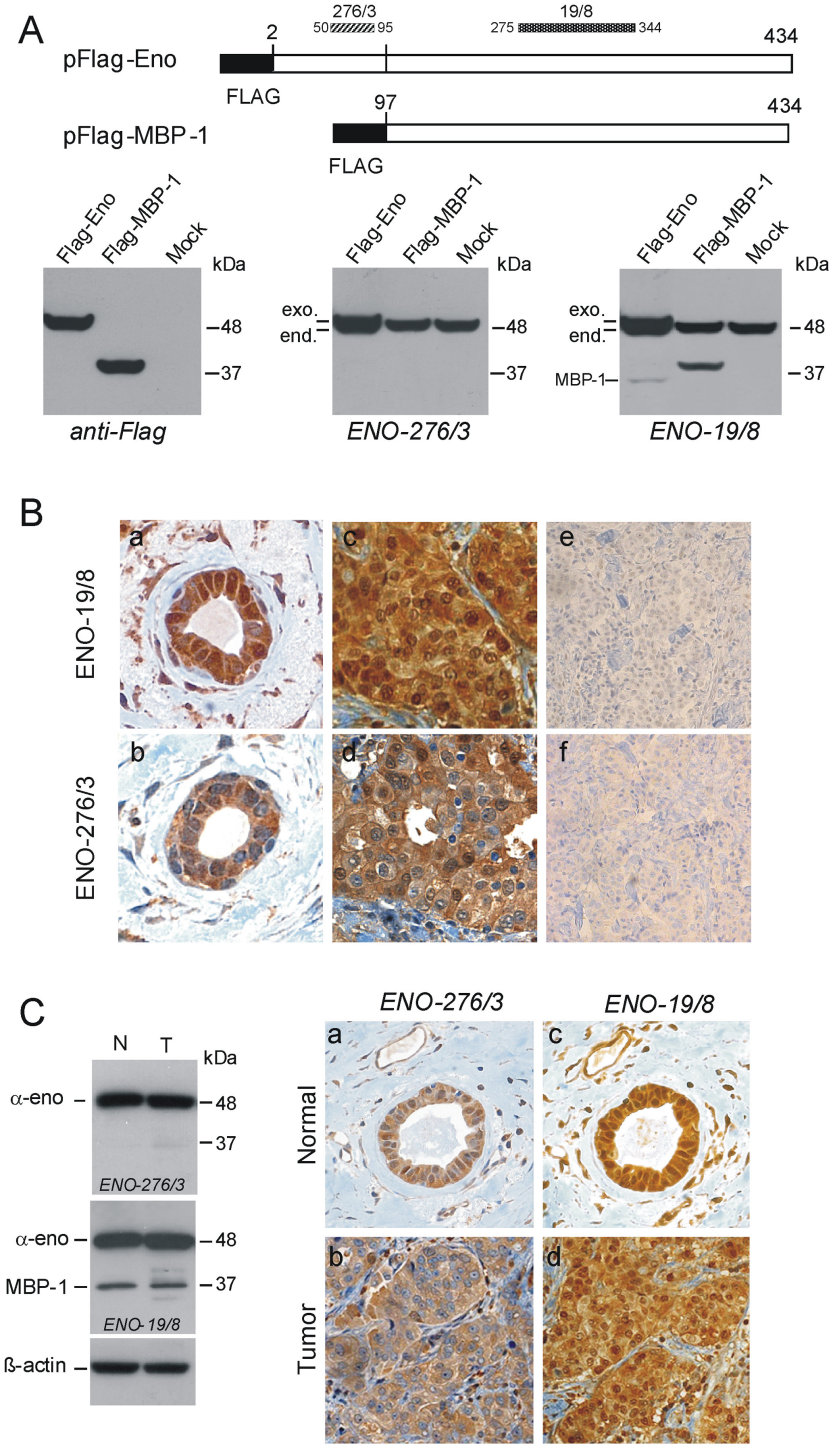
Monoclonal antibodies ENO-276/3 and ENO-19/8 specifically recognize α-enolase or both α-enolase and MBP-1. **A**. Top, schematic representation of Flag-tagged constructs used, black boxes indicate the position of the Flag epitope, and numbers indicate the first and last amino acid in each construct. Protein regions containing the epitopes recognized by ENO-276/3 and ENO-19/8 mAbs are indicated by striped and dotted bars respectively, numbers refer to corresponding amino acids in the α-enolase sequence (Swiss-Prot: P06733). Bottom, Western blot analysis of 293T cells transiently transfected with vectors expressing Flag-tagged α-enolase (Flag-Eno), or MBP-1 (Flag-MBP-1) proteins, compared to mock transfected cells (mock). Cell lysates (20 µg) were firstly probed with polyclonal anti-Flag antibodies and, after stripping, the same filter was reacted with anti-α-enolase ENO-276/3, and finally with ENO-19/8, as indicated. Molecular weight (kDa) are indicated on the right. Relative positions of endogenous (end.) and ectopically expressed (exo.) α-enolase and MBP-1 from transfected pFlag-ENO are indicated on the left. **B**. Representative immunohistochemical staining on serial sections of a normal mammary tissue (a and b) and an IDC tumors sample (c and d) from the commercial TMA. The anti-α-enolase mAbs ENO-19/8 specifically decorated both nuclei and cytoplasm, while ENO-276/3 gave an almost exclusive cytoplasmic staining. As a control, breast cancer sections (e and f) were immunoassayed with mAbs which had been previously coincubated and thereby blocked with antigenic peptides, as described in materials and methods. Magnification: 500× (a–d), and 200× (e and f). **C**. Representative Western blot and immunohistochemical analyses of normal and tumor tissues from IDC samples. Left, Western blot detection of α-enolase (α-eno) and MBP-1 proteins in total lysates (30 µg) from a breast tumor sample (T) and normal-matched tissue (N) using mAbs ENO-276/3 and ENO-19/8. Anti-β-actin antibodies were used as a control for loading. Right, immunohistochemical staining with mAbs ENO-276/3 and ENO-19/8 of serial sections of the tumor (b and d) and the corresponding normal breast tissue (a and c) from the same sample analyzed by Western blot. Magnification: 400×.

Epitope mapping was performed using full length and truncated recombinant α-enolase expressed in bacteria and synthetic peptides, results were confirmed by the ectopic expression of the wild type antigen and deletion mutants in human cells (data not shown). This wide screening allowed to select a monoclonal antibody, ENO-276/3, which recognizes an epitope within the NH2-portion of α-enolase (amino acids 50–95) and to map ENO19/8 binding site to an α-enolase region between amino acids residues 275 and 344 (Figure 2A).

Both ENO-19/8 and ENO-276/3 antibodies were finally tested on human embryonic kidney 293T cells transiently transfected with vectors expressing Flag-tagged α-enolase or MBP-1 (17, Figure 2A). Western blot analysis of protein lysates from 293T, which express very low levels of endogenous MBP-1, confirmed that ENO-276/3 antibody detects only the 48 kDa peptide, whereas ENO-19/8 antibody detects both the 37 kDa MBP-1 variant of α-enolase that lacks the first 96 aminoacids [10], [11] and the catalytic 48 kDa form, (Figure 2A). In optimal experimental conditions (80% transfection efficiency) a small amount of MBP-1 protein, arising from the alternative translation of the exogenous transcript for α-enolase, was detected (Figure 2A, right panel), as supported by the apparent molecular weight and the lack of reaction of both anti-Flag and ENO-276/3 antibodies, which indicates the absence of the NH2-terminal portion.

Reprobing the normal and breast tumor lysates shown in Figure 1A with ENO-276/3 antibody gave similar results to those obtained in transfected cells: α-enolase but not MBP-1 was detected in all the samples (data not shown).

Once defined the reactivity of the two monoclonal antibodies, immunohistochemistry was performed on serial sections of normal breast tissue and IDC from the commercial TMA, nuclear and cytoplasmic staining were detected using ENO-19/8 antibody (Figure 2B, a and c) and an almost exclusive cytoplasmic staining using ENO-276/3 antibody (Figure 2B, b and d). Furthermore, an antigenic peptide or the recombinant α-enolase-GST polypeptide completely blocked the immunohistochemical staining by ENO-276/3 and ENO-19/8 antibody, respectively (Figure 2B, e and f), confirming the specificity of the reactions.

Finally, to correlate Western blot results with immunohistochemical data and to exclude the possibility of different antibody reactivity in different experimental approaches, lysates and tumor sections from the same samples were analyzed with both antibodies. As expected presence of the 37 kDa MBP-1 variant consistently correlated with nuclear staining in histochemical analysis (Figure 2C).

Altogether these results indicate that the anti-α-enolase antibodies used in our study specifically recognize MBP-1 and/or α-enolase, and strongly support that the observed nuclear staining is indicative of antibody reactivity to MBP-1.

### Cytoplasmic α-enolase and nuclear MBP-1 expression: correlation to clinicopathological features of breast carcinomas

**Figure 3:**
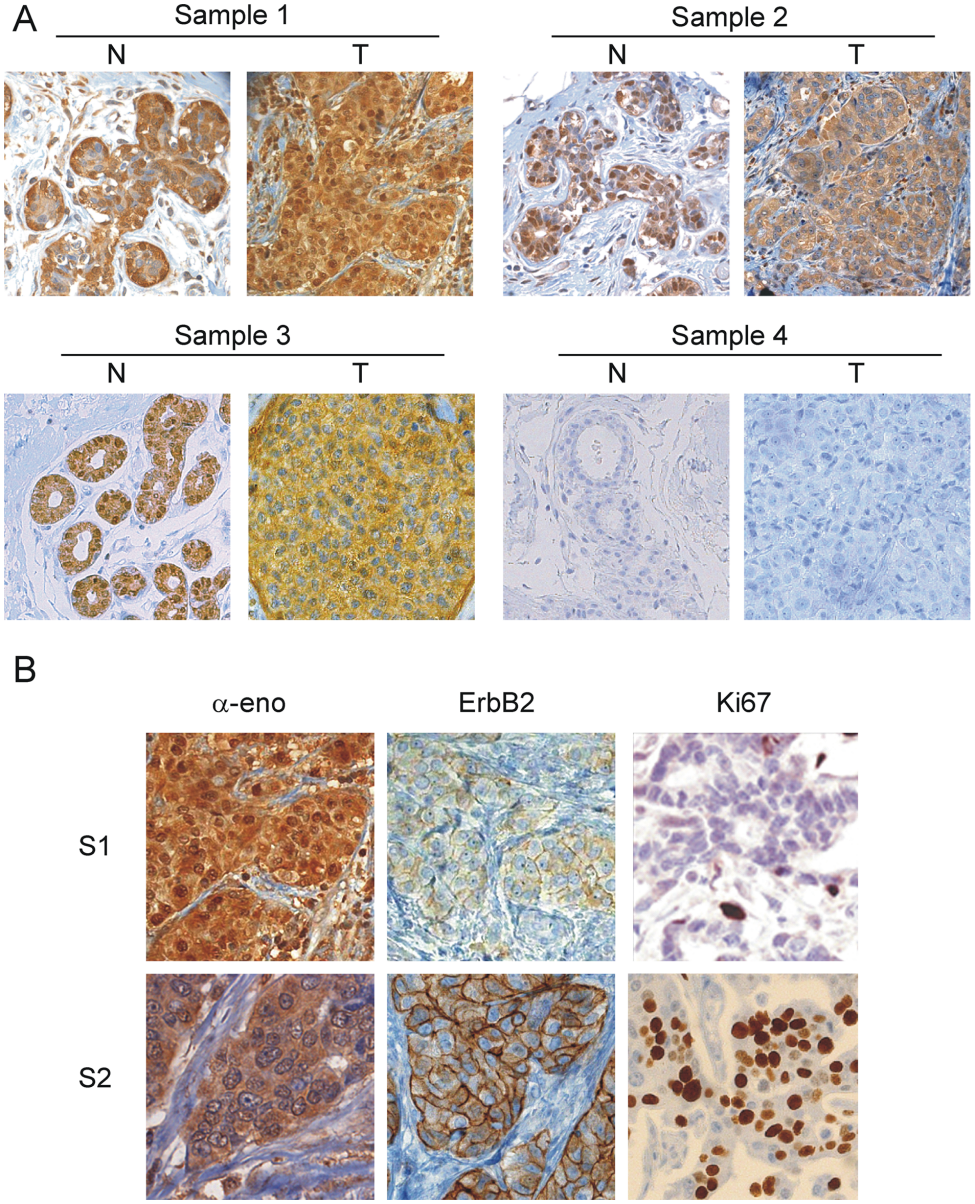
IDC immunohistochemical staining for α-enolase and nuclear MBP-1 revealed heterogeneity in breast cancer samples. **A**. Representative immunohistochemical staining of 3 out of the 177 IDC samples analyzed with mAbs ENO-19/8. Tumor sections (T) were compared with the corresponding normal breast tissue (N). All normal breast tissues showed nuclear MBP-1 expression and low expression of cytoplasmic α-enolase (sample 1–3). In sample 1 and 2, cytoplasmic staining of the invasive breast carcinoma (T) was stronger than in the matched normal breast tissues. In tumor samples 2 and 3, the loss of MBP-1 nuclear expression occurred. Sections of sample 4 were immunoassayed with antibodies blocked with a recombinant α-enolase polypeptide (see materials and methods). Magnification: 250×. **B**. MBP-1 nuclear staining correlated with ErbB2 and Ki67 expression. Immunohistochemical staining for α-enolase, ErbB2 and Ki67 in MBP-1-positive (S1) and MBP-1-negative (S2) tumors. Magnification: 500×.

To further investigate ENO1 gene products expression and localization in a larger number of samples, immunohistochemical analyses were performed using anti-α-enolase monoclonal ENO-19/8 as primary antibody. A series of 177 IDCs including for almost all the cases the adjacent normal breast tissue and the corresponding invasive cancer from the same patient were examined. Staining was observed in both the cytoplasm and nuclei of normal epithelium in 165 samples, which is consistent with the normal tissue microarray data. However, 66 out of 177 (37%) tumor specimens showed the loss or down-regulation of nuclear staining in the transition from normal epithelium to IDC. In Figure 3A are shown three representative IDC samples immunostained for α-enolase, one with strong nuclear staining (T, sample 1), and two with close to negative nuclear staining (T, sample 2 and 3).

As for the TMA, a significant number of normal and tumor samples showing nuclear staining were randomly selected and serial sections probed with the ENO-276/3 antibodies specific for full-length α-enolase (see Figure 2C for representative immunohistochemistry). As expected no nuclear staining was observed in all the samples examined, confirming that the nuclear immunoreactivity detected by ENO-19/8 is likely due to MBP-1, although it cannot be excluded its presence also in the cytoplasm.

Interestingly, additional immunohistochemical screening indicated that nuclear staining inversely correlates with ErbB2 and Ki67 expression in the large majority of the examined samples (see Figure 3B).

All the 177 tissue samples included in this study scored positively for the expression of cytoplasmic α-enolase and according to signal intensity they were graded as weak, moderate and strong: 31 (18%) had weak staining, 69 (39%) moderate and 77 (43%) very strong. With respect to the number of enolase labelled cells, at least 50% cytoplasmic labeling was observed in all cases. In addition, α-enolase expression was predominantly detected in the cytosol of tumor cells, whereas a weak staining was observed in stromal and myoepithelial cells. All normal breast tissues showed moderate expression of cells labelled α-enolase, whereas stronger expression was observed in the paired tumor samples, however, no significant correlation between expression of the protein and clinicopathological characteristics of tumors or patients outcome was observed.

To grade tumors relatively to MBP-1, a cut-off value for nuclear MBP-1 expression was chosen on the basis of a measurement of heterogeneity using the log-rank test statistical analysis with respect to disease-free survival. An optimal cut-off value was identified as 20% of stained nuclei, and it was used to define tumors as MBP-1-negative (≤20%, absent/low expression) or MBP-1-positive (>20%, medium/high expression). Statistical analyses were done to examine the correlation between nuclear MBP-1 expression, as detected by immunohistochemical staining, and the clinicopathological characteristics of breast cancers. As shown in Table 1, in patients with IDC no correlation was found between the expression levels of nuclear MBP-1 and patient age, tumor size, death by disease or estrogen and progesterone receptors expression levels. In contrast, MBP-1 expression strongly correlated with the node status (*P* = 0.0002), tumor grade (*P*<0.0001) and expression levels of ErbB2 (*P* = 0.0001) and Ki67 (*P* = 0.0096) proteins. These data were further confirmed by employing the Spearman correlation analysis to test the correlation between MBP-1 expression and the clinicopathological features. As shown in Table 2, the Spearman correlations of nuclear MBP-1 expression levels to node status, tumor grade, recurrence of metastasis, and the expression of ErbB2 and Ki67 were: −0.233 (*P*<0.0001), −0.289 (*P*>0.0001). −0.226 (*P* = 0.022), −0.189 (*P* = 0.005), and −0.220 (*P* = 0.001), respectively. Taken as a whole, the expression of MBP-1 was negatively correlated with clinical staging.

**Table 2:** Spearman correlation analysis between MBP-1 and clinicopathological features.

Variables	MBP-1 nuclear expression	
	Sperman correlation (*p*)	
Node status	−0.233	(<0.0001)
Tumor grade	−0.289	(<0.0001)
ErbB2 status	−0.189	(0.005)
Ki67 status	−0.220	(0.001)
Recurrences	−0.226	(0.022)

### Nuclear MBP-1 Expression is associated with Recurrence-Free Survival in Breast Cancer

**Figure 4:**
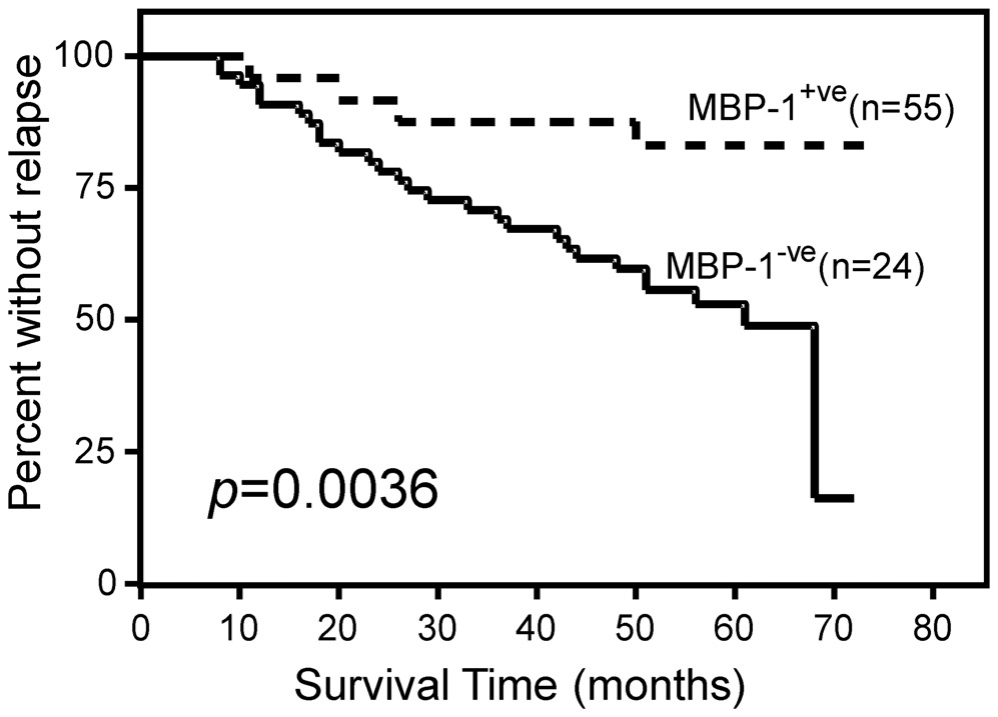
Survival analysis of IDC patients according to MBP-1 expression. Kaplan-Meier curves for disease-free survival (DFS) in 77 IDC patients relative to nuclear MBP-1 positive (+^ve^) or negative (−^ve^) expression. The absence or low expression (MBP-1^−ve^) of nuclear MBP-1 was significantly associated with recurrence (*P* = 0.0036 by log-rank test).

To test nuclear MBP-1 expression as a potential predictor of survival it was correlated to patient's status in a statistically significant number of IDC. The loss of nuclear MBP-1 expression in breast cancer tissues significantly predicted local recurrence. In a five year follow-up, MBP-1-positive expression significantly correlated with a 92% local recurrence-free survival. In contrast, for IDC samples that showed the loss of MBP-1 expression, recurrence-free survival fell to 54% at five year after surgery (*p* = 0.0036) (Figure 4).

A multivariate analysis was performed, according to Cox regression model, for disease-free survival, including as covariates MBP-1, ErbB2 expression and lymph node status. These last two factors were chosen based on the results of the univariate analysis (data not shown) and because they are known to influence survival of breast cancer patients. Nuclear MBP-1 expression was found to be an independent favourable prognostic indicator for disease-free survival (Table 3). Thus, nuclear MBP-1-negative staining, revealed by immunohistochemistry, could be an important marker to select breast cancer patients for increased vigilance in follow-up and adjuvant therapy.

**Table 3:** Multivariate analysis for disease-free survival.

Variable	Hazard ratio	*P* Value	95% CI
MBP-1^a^	0.319	0.047	0.6201–1.7080
ErbB2^b^	1.607	0.211	0.3649–1.1251
Node status^c^	1.994	0.68	0.3633–1.1203

a MBP-1 positive vs MBP-1 negative.

b ErbB2 positive vs ErbB2 negative.

c Node positive vs Node negative.

CI, confidence interval.

## Discussion

The present study provides novel and important findings on the expression and subcellular distribution of α-enolase and MBP-1 proteins in normal breast tissue and tumors.

The glycolytic enzyme α-enolase and the transcriptional repressor MBP-1 arise from the ENO1 gene and differ for the first 96 amino acids residues, not present in the MBP-1 variant.

To date several studies have reported α-enolase immunoreactivity in cytoplasm, nucleus and cell membrane. The predominantly cytoplasmic localization of the glycolytic enzyme α-enolase has been widely demonstrated in cell lines by subcellular fractionation, while we and others have shown nuclear accumulation of exogenous MBP-1 compared to the full length protein [10], [16], [18], [27]. Previous histochemical studies conducted on tumor samples and normal tissues have reported three expression patterns of ENO1 gene products: cytoplasm, nuclei and both nuclei and cytoplasm [23], [27]. To date, only one study has reported the identification of α-enolase as a nuclear protein in human adrenal cortex [33].

Our data, based on the use of highly specific monoclonal antibodies, show that: i) nuclear staining in normal and tumor breast tissue is mainly due to MBP-1 expression; ii) nuclear MBP-1 is a common feature of normal breast tissue; iii) MBP-1 expression is associated with good survival of patients with IDC. Thus, MBP-1 is the first transcriptional repressor whose expression has the potential to predict a good outcome in breast cancer.

Our finding of a frequently decreased expression of MBP-1 protein in IDCs supports a role for MBP-1 as a tumor suppressor and raises questions concerning the mechanism responsible for its loss in these tumors. It is interesting to note that the ENO1 gene, encoding α-enolase and MBP-1, is located in the distal short arm of chromosome 1 [34] within one of the most frequent regions of loss of heterozygosity (LOH) observed in node negative breast cancers with poor prognosis [35], as well as brain tumors and hematopoietic neoplasms, suggesting that MBP-1 could be a candidate tumor suppressor in that region. Studies on neuroblastoma seem to rule out that mutations inactivating the ENO1 gene are involved in controlling MBP-1 expression [28]. Our preliminary studies on LOH of the chromosome region surrounding the ENO1 locus (1p36.3) in the IDC samples did not revealed any correlation between 1p36.3 LOH and MBP-1 loss of expression. In addition, we did not find a correlation between nuclear MBP-1 expression and cytoplasmic α-enolase levels in IDC. These data suggest that MBP-1 expression is regulated independently from the expression of the catalytic cytoplasmic isoform.

Recently, it has been reported that altered glucose concentration [36] and hypoxia [37] result in the differential translation of α-enolase/MBP-1-encoding mRNA in the MCF-7 breast cancer cell line, strongly suggesting that posttranscriptional mechanisms might play a role in the regulation of MBP-1 expression in breast cancer cells. Studies are ongoing to identify the mechanisms involved in MBP-1 down-regulation in these tumors.

Ectopic expression of MBP-1 has been reported to suppress tumorigenicity of breast cancer [22], and prostatic cancer [38] cell lines in nude mice, suggesting the significance of MBP-1 expression as an indicator of favorable prognosis and a protective role of MBP-1 against cancer progression. Recently, it has been shown that MBP-1 overexpression results in the modulation of MMP-2 expression, the inhibition of *in vitro* angiogenesis and the regression of primary and metastatic breast tumor growth in an immunocompetent mouse model, [29].

In agreement with these findings, in our study on human primary tumors, nuclear MBP-1 expression was associated with good outcome, further supporting that the disregulation of MBP-1 expression may play an important role in promoting cancerogenesis and the progression of breast cancer.

In conclusion, we present evidence that nuclear MBP-1, which inversely correlated with ErbB2 and Ki67 protein expression, could be used as an independent prognostic variable of clinical outcome and may represent a potential target for therapy in primary IDC patients. This suggests that patients with IDC, who show the loss of nuclear MBP-1, are at higher risk of recurrence and death from disease and may eventually benefit from more aggressive adjuvant therapy. However, the reliability of MBP-1 as a potential marker in the routine clinical assessment and management of patients with IDC deserves further evaluation in long-term follow-up studies on a larger number of cases.

In addition, functional studies are required to elucidate the role of MBP-1 and its downstream and upstream regulatory pathways in the pathogenesis of IDC.
